# The Diversity of Venom: The Importance of Behavior and Venom System Morphology in Understanding Its Ecology and Evolution

**DOI:** 10.3390/toxins11110666

**Published:** 2019-11-14

**Authors:** Vanessa Schendel, Lachlan D. Rash, Ronald A. Jenner, Eivind A. B. Undheim

**Affiliations:** 1 Centre for Advanced Imaging, the University of Queensland, St. Lucia, QLD 4072, Australia; v.schendel@uq.edu.au; 2 School of Biomedical Sciences, the University of Queensland, St. Lucia, QLD 4072, Australia; l.rash@uq.edu.au; 3 Department of Life Sciences, Natural History Museum, Cromwell Road, London SW7 5BD, UK; r.jenner@nhm.ac.uk; 4 Centre for Biodiversity Dynamics, Department of Biology, Norwegian University of Science and Technology, 7491 Trondheim, Norway; 5 Centre for Ecological and Evolutionary Synthesis, Department of Biosciences, University of Oslo, P.O. Box 1066 Blindern, 0316 Oslo, Norway

**Keywords:** Venom diversity, venom metering, venom optimization, venom gland, predation, defense, toxin function

## Abstract

Venoms are one of the most convergent of animal traits known, and encompass a much greater taxonomic and functional diversity than is commonly appreciated. This knowledge gap limits the potential of venom as a model trait in evolutionary biology. Here, we summarize the taxonomic and functional diversity of animal venoms and relate this to what is known about venom system morphology, venom modulation, and venom pharmacology, with the aim of drawing attention to the importance of these largely neglected aspects of venom research. We find that animals have evolved venoms at least 101 independent times and that venoms play at least 11 distinct ecological roles in addition to predation, defense, and feeding. Comparisons of different venom systems suggest that morphology strongly influences how venoms achieve these functions, and hence is an important consideration for understanding the molecular evolution of venoms and their toxins. Our findings also highlight the need for more holistic studies of venom systems and the toxins they contain. Greater knowledge of behavior, morphology, and ecologically relevant toxin pharmacology will improve our understanding of the evolution of venoms and their toxins, and likely facilitate exploration of their potential as sources of molecular tools and therapeutic and agrochemical lead compounds.

## 1. Introduction

Venoms are biochemical arsenals containing mixtures of bioactive compounds that consist of salts, small molecules, and proteins and peptides, which are commonly referred to as toxins [1]. These toxins function, individually or synergistically, by targeting essential components of normal physiological and signaling processes, often with great potency [2]. Consequently, toxins have been an important source of molecular tools for dissecting physiological processes [3], and lead molecules for the development of drugs targeting a range of conditions such as chronic pain [4], diabetes [5], cancer [6], stroke [7], and autoimmune disease [8]. There are currently seven venom-derived molecules that have been turned into commercial drugs, including a cone snail toxin-derivative for treatment of chronic pain (Prialt^®^, AstraZeneca), and a Gila-monster toxin-derivative for treatment of type-II diabetes (Byetta^®^, AstraZeneca) [9]. Considering that individual venoms may contain up to hundreds of unique compounds [10] and that the venoms from even comparatively well-studied lineages remain largely unexplored [11,12], there is an enormous unexplored natural library of bioactive compounds contained in animal venoms.

**Figure 1:**
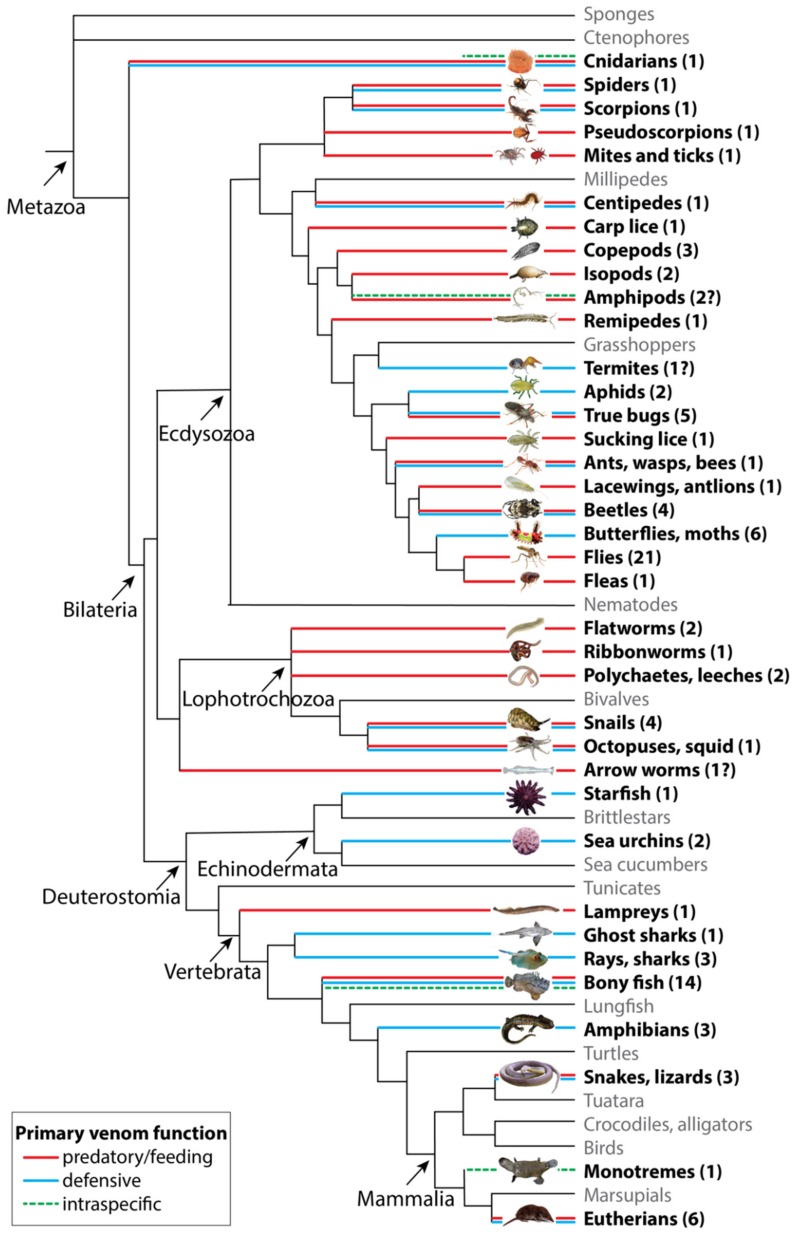
Taxonomic diversity and the main primary functions of venom. A pruned and schematic phylogenetic tree of venomous animals modified after Casewell et al. [23] illustrating the frequency with which venoms have evolved within the animal kingdom. Colored branches highlight venomous lineages, with red branches indicating a predatory/feeding venom function, blue branches indicating a defensive function and dashed green branches indicating a role in intraspecific competition. Taxa for which no direct support of their venomous nature could be found are indicated with a question mark. For an exhaustive list of venomous lineages see Table S1. Arthropod phylogeny follows that of Giribet and Edgecombe [24].

In addition to their value in applied research, venoms are interesting models for understanding the biology and evolution of adaptive traits and the functional evolution of proteins. Venom systems have evolved independently more than 100 times in an extremely wide range of taxa that includes at least eight separate phyla (see Figure 1). In each of these lineages, toxins—which are usually primarily proteins and peptides—have evolved from non-toxin ancestral proteins and peptides. In addition, many of these proteins and peptides have been convergently recruited to venomous functions in different lineages. CAP (cysteine-rich secretory proteins (CRISP), Antigen 5 (Ag5), and Pathogenesis-related (P R-1)) proteins, for example, can be found in the venoms of snakes, cephalopods, cone snails, several insects, scorpions, spiders and centipedes [13]. Thus, venoms—and the toxins they contain—should provide excellent models for studying processes of adaptive evolution using powerful comparative approaches [14].

The potential of toxins as models for studying molecular adaptive evolution is currently limited by the fact that the function(s) of individual toxins remains poorly known. Historically, most venoms have been studied either to understand their damaging effects on humans in order to prevent morbidity or mortality, or in the context of biological tool discovery. Venoms and toxins have thus been primarily characterized using human and other mammalian tissues and receptors. An exception to this is the targeted screening of venoms as sources of novel insecticides [15,16,17,18], which typically rely on a limited number of pest or pest-related insect species. Nevertheless, most toxin pharmacology is based on species that are ecologically irrelevant and/or represent only a fraction of the taxonomic diversity targeted by the venomous animal in nature, which likely affects the perceived ecological role due to differences in toxin activity in the model and the ecologically relevant species [19,20,21]. In addition, ecological contexts are normally not considered in the targeted screening or clinically-relevant studies that tend to dominate toxinology. Although most venoms function in predator-prey interactions (predation as well as defense), they fulfill other and/or additional functions as well (Table 1). However, what these functions are remains relatively poorly understood for the vast majority of venoms. We therefore know little about the behaviors associated with venom use, the effects of venom toxins on their natural targets, and the functions played by venom beyond predator-prey interactions. This lack of knowledge limits our ability to identify adaptive molecular changes, which is not only important for understanding how venoms evolve, but can also limit its utility in applications such as protein engineering [22].

To understand the evolution of venoms and their toxins, and better fulfill their potential as evolutionary models and molecular toolkits, there is a need to investigate both the behavioral aspects of venom use and the functional morphology of the whole venom apparatus—aspects of venom research that have been largely neglected in the past. Here, we summarize the current state of knowledge on the taxonomic and functional diversity of animal venoms and their toxins and review how individual animals are able to achieve these functions through various levels of behavioral control that regulate and modulate venom delivery. We then examine how venom system morphology facilitates and constrains these levels of control, and how this, in turn, affects the molecular evolution and pharmacology of toxins. Our findings highlight the need for holistic approaches to studying venoms, and will hopefully encourage more studies that include behavioral, morphological, and molecular aspects of venoms in order to help fill this black box in venom biology.

## 2. The Functional Diversity of Venoms

In order to understand the function of venoms and their toxins, it is important to know when and for which reason venom is used. Different species use venom for different purposes, with the most obvious and common purposes being predation and defense. For example, snakes, spiders, scorpions, and centipedes use their venom to immobilize or kill prey for consumption (Figure 1), while aculeate hymenopterans (e.g., bees and ants) and most venomous fish mainly use their venom to defend themselves against potential predators (Figure 1) [1,25]. A survey of all known independently evolved venomous lineages suggests that the primary function of venom in most of these is indeed to facilitate feeding, either through prey incapacitation or by enabling some form of ectoparasitism, often involving hematophagy (65 *versus* 40 defense and 4 intraspecific competition; Figure 1, Table S1, based on a conservative estimate). Although this number is skewed by toxinologically megadiverse groups such as the flies (Diptera), where venom has evolved to facilitate feeding 21 times, this is also the case for the number of defensive lineages, of which 32.5% (13 of 40) are bony fish (Osteichthyes).

However, venom can be used for more than just feeding, predation and defense (Table 1). Platypus males use a venomous spur on their hind legs to compete against other males during the mating season [26]. Male scorpions in several taxonomic groups apparently inject a small amount of venom into the female’s body during sexual encounters [27], although the purpose behind this so-called “sexual-sting” is not yet understood. Tawny crazy ants (*Nylanderia fulva*) use their venom to neutralize fire ant venom [28], and venoms of several different taxa have antimicrobial attributes [29]. Moles and shrews are thought to use their venom to store food—they inject venom into prey organisms to paralyze, but not kill them, and leave the immobilized prey in their burrow for later consumption [30]. Similarly, spider wasps such as tarantula hawk wasps rapidly paralyze but do not kill spiders so they can lay an egg on them [31,32], while other ectoparasitoid wasps, such as the cockroach-hunting jewel wasp (*Ampulex compressa*), use their venom to suppress the escape response of cockroaches without paralyzing them [33]. In the case of the jewel wasp, the wasp stings the cockroach in the brain to create a “zombie”-cockroach that is willingly guided back to the nest of the wasp, despite being several times heavier than the wasp herself [33]. While ectoparasitoid wasps use their venom to preserve food for their young, endoparasitoid wasps use their venom to transform organisms into a new habitat, or nurseries, for their offspring to live in and feed on when they hatch [34]. To achieve this, endoparasitoids such as *Nasonia vitripennis* inject venom into the host prior to oviposition to selectively suppress parts of the immune system, arrest development, and manipulate the internal nutritional environment [35,36].

Venoms can clearly serve very different functions in different species, but they are also used for more than just one purpose by many, if not most, species. For example, individuals of numerous lineages, including spiders, scorpions, and centipedes do not only inject a paralyzing venom into prey organisms but also use the same venom-delivering structures to defend themselves against potential predators via defensive bites and stings (Figure 1). Furthermore, some snakes (including spitting cobras, scorpions, wasps, assassin bugs, spiders (e.g., the green lynx spider (*Peucetia viridans*) and spitting spiders in Scytodidae), and ants (that usually inject venom into prey) spray venom in defensive situations instead of injecting it [37,38,39,40,41]. Spitting spiders (Scytodidae) are even able to spit a mixture of silk and toxic glue from their venom apparatus in order to capture prey [42]. Thus, a venomous animal may have different venom components that have evolved to play different ecological roles, e.g., painful defensive toxins, paralyzing predatory toxins, behavior-altering neurotoxins, and even stimulating toxins that are used during copulation. Venoms are, in this regard, essentially ecological Swiss army knives, with multiple components performing one or several functions that together make up a highly complex adaptive trait. To date, only a few studies have investigated whether, for example, predatory and defensive venoms of the same species actually differ in composition.

Functionally distinct venom toxins are perhaps best characterized in sea anemones. As cnidarians, sea anemones do not have a centralized venom system but are instead covered with venom producing cells called nematocytes that contain the venom-delivering nematocysts, as well as toxin-secreting epidermal gland cells [43]. This means that the ecological function(s) of toxins can be inferred from their distribution across the sea anemone functional anatomy, such as tentacles (predation, sometimes also defense), mesenterial filaments (digestion), gametes (protection of eggs), acrorhagi (intraspecific competition), and acontia (defense) [44,45,46,47,48]. In addition to these functionally distinct tissues and associated toxin mixtures, sea anemones even have different types of venom during different life stages—defensive venom in the early stage (planulae) and defensive as well as predatory venom in later stages (polyp) [49]. Unlike sea anemones and other cnidarians, however, venom systems of the majority of other venomous animal lineages consist of a single or paired set of venom-producing glands connected to a set of delivery structures. Injecting a mixture of toxins with different ecological functions at once appears to contradict the widely accepted idea that animals optimize their venom use and composition to minimize energetic expense [50].

## 3. Venom Modulation

**Figure 2:**
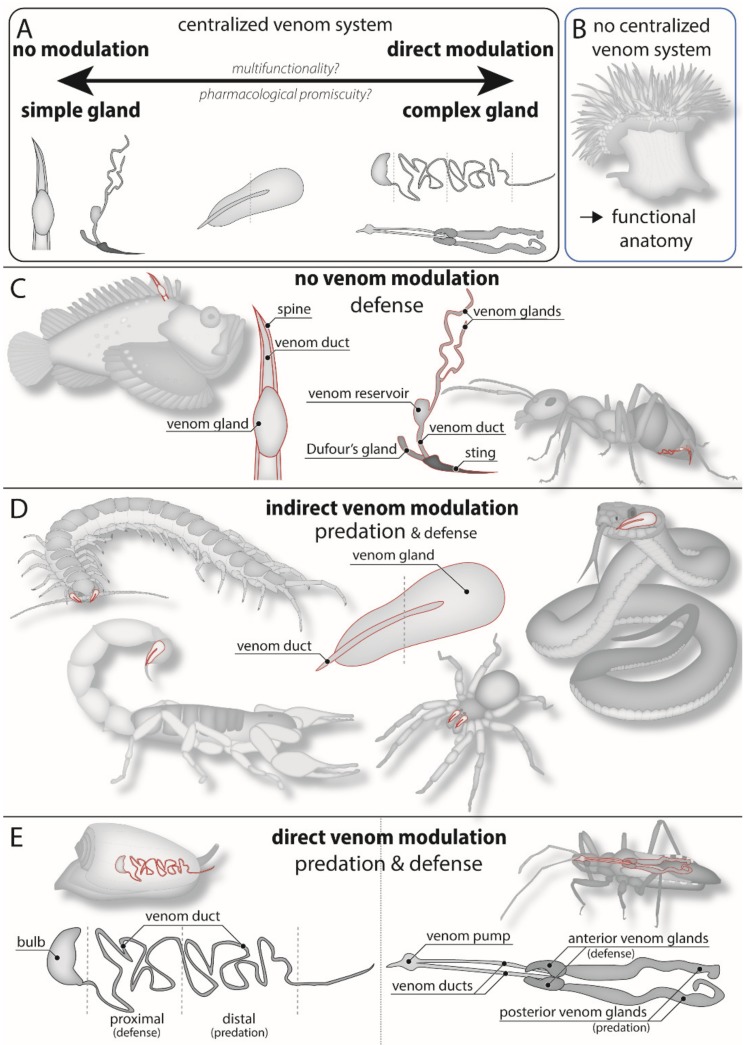
Examples of morphological constraints on the regulation of venom secretions. (**A**) Species with complex venom glands and heterogeneous distribution of venom components are more likely to be able to qualitatively modulate venom compared to species with simple venom glands. This ability may, in turn, be related to the pharmacological properties of toxins in the venom (see below). (**B**) Sea anemones do not possess a centralized venom system (venom gland). Instead, the functions of toxins can be inferred from the sea anemone’s functional anatomy. (**C**) Ants and venomous fish possess rather simple venom glands with only a few different components and are not able to modulate venom secretion. They use their venom to defend themselves against potential predators, but in the case of the ants also to incapacitate prey. (**D**) Snakes, spiders, and centipedes are also thought to be able to modulate venom composition as venom components are stored heterogeneously throughout the gland. This may enable indirect qualitative venom modulation similar to that which has been demonstrated in scorpions, which possess a roughly similar overall venom gland morphology as snakes, spiders, and centipedes. (**E**) Cone snails and assassin bugs are able to directly modulate venom composition. They achieve this due to complex venom gland morphology with distinct compartments for predatory and defensive venom components.

One solution to the apparent contradiction between venom optimization and the non-overlapping functions of toxins is to regulate, or modulate, the amount or composition of the venom secreted according to the ecological context. Animals with centralized venom systems may regulate secreted venom either quantitatively or qualitatively (Figure 2). That is, they either regulate the amount of injected venom—also often referred to as “venom metering” [50]—or the biochemical composition of the venom. These abilities are not mutually exclusive but are in many cases likely to be hierarchically interdependent in that quantitative regulation is a prerequisite for qualitative modulation, but not vice versa. They do, however, have vastly different implications for understanding the ecology and evolution of venoms and their toxins.

### 3.1. Quantitative Regulation of Venom

It is relatively well known that some venomous animals are able to meter venom according to different situations [50,71]. However, if, how, and why venomous animals meter venom expenditure is poorly understood. According to the widely accepted venom optimization hypothesis [50,71], one reason for only secreting a certain amount of venom is rather simple—venom production is energetically costly and thus venom should not be wasted. However, whether the production of venom really represents a substantial metabolic cost is still a matter of debate [50,72], and very few studies have actually investigated the metabolic costs of venom production. While most of these studies did demonstrate a higher metabolic rate after venom expenditure compared to a resting state [73,74,75,76,77,78], it has also been reported that in comparison with, for example, molting or food digestion, the metabolic costs of venom expenditure (at least for snakes) seem fairly low [72,73,75]. The energetic costs of venom production may therefore not be as high as often suggested.

Another possible rationale for not wasting venom relates to the multi-functionality of most venoms. Overspending venom on one purpose represents a needless depletion of valuable tools for other purposes. In other words, unnecessarily depleting venom reservoirs in a defensive situation also means that there has been an unnecessary depletion of not just defensive but also predatory toxins, or *vice versa*. It can take up to several days or weeks for some venom components to be regenerated [50,71,74,77,79,80] and during that time of regeneration, the venomous animal is likely to be both more vulnerable to predators or competitors and less able to capture prey. The combination of the need to escape predators and capture prey likely results in strong selection against the “frivolous” expenditure of venom. This hypothesis does obviously not discount the contribution of reducing the metabolic expense of venom use, which could still drive the evolution of regulation of venom secretion for different purposes—perhaps explaining why, for example, western diamondback rattlesnakes inject more venom in defensive than predatory strikes against same-sized prey [81].

There are different ways of how animals can regulate venom expenditure and thus save venom for when it is really needed, such as minimizing venom use in low-threat situations or for small prey organisms for which superior physical strength is sufficient. Indeed, in some situations, bites and stings are not used at all, such as when scorpions crush small prey with their pedipalps [82], giant centipedes use their powerful ultimate (hindmost) legs to deliver defensive mock “bites” [83], spiders use silk to immobilize prey [84], and venomous snakes use constriction to subjugate prey [85]. Defensive dry bites or dry stings (those in which no venom is apparently injected) are also quite common in venomous animals such as snakes, scorpions, and spiders [84,86,87]. If the animal does decide to use venom, the site of injection into the prey or predator’s body also seems to be of importance, and there is evidence that venomous animals preferably inject venom into parts of the prey’s body where it is most efficient [88,89]. The prairie rattlesnake *Crotalus viridis* and the centipede *Scolopendra subspinipes mutilans*, for example, seem to prefer to attack the head region rather than the abdomen, and centipedes have even been observed to reorient prey to be able to inject venom into the preferred body part [88,89]. It has also been observed that depending on, e.g., prey size, the venomous animal injects only once versus several times [84], and thereby carefully regulates the total amount of venom spent [90].

In addition to regulating whether or not, or the number of times, venom is secreted, there is evidence that many venomous animals are able to regulate the amount of venom that is delivered in a single sting or bite [50,71,79,86,89,91]. Spiders, snakes, and scorpions generally appear to inject more venom into prey organisms that are large, difficult to handle, or not very susceptible to their venom, compared to small, easy to handle, and susceptible prey, where they only inject low amounts of venom [50,81,89,92,93]. Furthermore, prey preference may depend on how much venom is available [50,94]. When not much venom is left, small and easy-to-handle prey is preferred, while bigger prey that might need more venom to be subdued is avoided. Thus, it seems that many venomous animals have an awareness of their venom reserves and deliberate control over how much they inject.

### 3.2. Qualitative Modulation of Venom

While quantitative regulation of venom reduces unnecessary venom expenditure, it does not directly address the apparent contradiction of why many venoms that play multiple roles contain such an abundance of functionally non-overlapping toxins. To this end, several species have been shown to be able to qualitatively modulate their venom according to the ecological context [95,96,97,98]. The South African fat-tail scorpion, *Parabuthus transvaalicus*, possesses a transparent potassium-rich “pre-venom” which differs from subsequently secreted protein-rich milky venom [97]. The potassium-rich pre-venom is secreted first and is likely energetically “cheaper” to produce and faster to regenerate than the later secreted protein-rich milky venom [97]. While pre-venom causes pain and is probably used for defense, the protein-rich venom should be highly efficient for predation [97]. It has been shown that scorpions are able to meter venom in defense situations and choose between using dry stings, pre-venom, and venom, with the protein-rich venom only being used in high-threat situations [86,99,100]. Furthermore, it has been proposed by several authors that transparent pre-venom is also used for the sexual sting [27,97]. However, it has yet to be demonstrated whether scorpions are able to inject venom that differs in peptide toxin composition.

Proteomic and transcriptomic analyses combined with multimodal imaging revealed that the assassin bug *Pristhesancus plagipennis* produces two different venom cocktails in two distinct lumens of the main venom gland (Figure 2e) [96]. Proteins and peptides produced in the anterior gland lumen are secreted upon harassment, but much less so upon milking by electrostimulation, are not paralytic in insect models (*Lucilia cuprina* and *Acheta domesticus*), and are therefore thought to have a defensive role. In contrast, proteins and peptides obtained by electrostimulation are produced in the posterior gland lumen and are likely to be used for predation as they potently paralyze and kill prey insects [96]. Similar functionally distinct compartments have been found in the main venom glands of other assassin bugs [101,102], although neither the role nor functional specialization of these compartments appears to be shared across all assassin bugs [103,104]. Interestingly, this venom system architecture is also shared with the homologous salivary glands of non-venomous non-heteropteran hemipterans, such as cicadas, where they are thought to perform different roles while feeding on plant sap [105], supporting the idea that this distinction is a morphological pre-adaptation that enables qualitative venom modulation.

A similar scenario has been described for cone snails (*Conus* spp.), some of which have been shown to be able to rapidly switch between predatory and defensive venom. In this case, the defensive and predatory venoms are produced in different parts of the long venom gland (“duct”), which is expelled using a venom “pump” situated distally to the venom-injecting harpoon (Figure 2e) [95]. Defensive venom from *C. geographus* contains paralytic toxins that block neuromuscular receptors and can be lethal to humans, while the predatory venom contains mainly prey specific toxins with little to no known effect on humans [95]. Some cone snail species have thinner shells than others and might thus have evolved highly-potent defensive venoms to better protect themselves against potential predators [106]. Interestingly, it has also been hypothesized that the evolution of these distinct defensive venoms in ancestral worm-eating cone snails facilitated a switch in diet that in turn drove the enormous functional radiation of conotoxins. Instead of using their venom to defend themselves against fish and molluscs, ancestral mollusc- and fish-hunting cone snails started to use their venom to prey on their former predators instead [95].

Although qualitative venom modulation has so far only been investigated in a few species, it seems likely that as more studies are carefully designed and carried out, more venomous animals will be revealed to be able to modulate venom expenditure and/or composition in some way (see below). A major challenge to this endeavor remains the ability to obtain natural venom secretions, i.e., venom that is not obtained by chemical (e.g., pilocarpine) or physical (e.g., electrostimulation or massaging) stimuli of the venom apparatus. As a result, and despite the compelling evidence for deliberate control over venom secretion summarized above, very little is known about how prevalent qualitative modulation is across the myriad of venomous lineages, and how this ability is actually achieved. In order to answer these questions, detailed knowledge of venom secreting behavior, the venom apparatus, and its functional morphology are required.

## 4. Morphological Constraints on Venom Modulation

While it seems likely that there are several venomous lineages that have the ability to qualitatively regulate venom, it is certainly not a universal feature of venom. One of the key determinants of the ability to modulate venom is the venom gland and delivery system anatomy—or venom system functional morphology. It follows therefore that venom system morphology is crucial to understanding the evolution of venoms and toxins. Venom apparatus anatomy and organization differ dramatically between venomous lineages, and this greatly affects how venom is secreted and to which extent it can potentially be modulated. For example, cone snails and assassin bugs possess complex venom glands with different compartments and structures for venom secretion and expulsion (Figure 2e) [95,96]. In both these cases, toxin secretion and venom expulsion are carried out by morphologically separate structures. Although nothing is known about the neuronal innervation of these complex venom systems, and their mechanisms of modulation remain largely speculative, this morphological segregation of secretion and expulsion is likely a prerequisite for the differential secretion of toxins, or direct qualitative modulation of venom (Figure 2e).

Unlike cone snails and assassin bugs, scorpions, at least observed so far, appear to be able to only indirectly influence venom composition by metering the amount of venom secreted. For example, while *P. transvaalicus* secreted distinctive pre-venom and proteinaceous venoms during “controlled” sting series, where venom is incrementally secreted through serial relatively minor stings, this is not necessarily the case in high-threat situations. During these situations, *P. transvaalicus* can elicit a peculiar defensive venom-spraying behavior, where the defensive sprayed venom is white in color [86,99]. This suggests that either the sprayed volume of venom is greater than the available pre-venom volume, resulting in the secretion of otherwise perhaps largely non-defensive proteinaceous venom, or that the scorpion may indeed have qualitative control over venom secretion. However, scorpion venom glands are relatively simple compared to those of assassin bugs and cone snails—all venom is secreted into a single branch-like lumen where the components are mixed and expelled through a single duct. While the venom glands of at least some species, such as *Centruroides sculpturatus*, have folded secretory epithelia that appear to be innervated by neurons, the functions of these neurons remain unknown [107]. Similar innervation has also been observed in spiders and snakes [108,109,110,111], where they have been shown, at least in snakes, to be involved in the venom regeneration process. This may also be the case in the observed neuronal innervation of the scorpion venom gland, although we cannot discount the possibility that scorpions may exert greater control over their venom secretion than their venom gland morphology suggests. Nevertheless, the lack of any obvious morphological (pre-)adaptations for direct qualitative venom modulation suggests that scorpions are able to modulate venom composition only indirectly through the displacement of toxins that are non-uniformly stored throughout the venom gland, that is indirect qualitative modulation of venom (Figure 2d).

While it is likely that there are more venomous lineages that possess the ability to directly modulate their venom, the indirect qualitative modulation of venom is probably a more widespread phenomenon. For example, snakes, spiders, and centipedes possess comparably simple venom glands (mainly consisting of one venom gland and duct with no separate expulsion mechanism), but they are thought to be able to modulate venom composition due to non-uniform distribution of toxins in their venom glands [27,97,98,112,113] (Figure 2d). This heterogeneous distribution of toxins along the direction of secretion is likely a prerequisite for qualitative venom modulation by toxin displacement, but likely represents lower evolutionary constraints or pre-adaptive requirements than the complex structures required for direct modulation of venom. It also highlights the importance of considering toxin production and storage when investigating venom system functional morphology, for example as determined by mass spectrometry imaging or in situ hybridization on venom gland sections [112,114].

While modulation of venom is likely to be more common among venomous animals than is currently appreciated, some venomous animals possess venom glands that do not seem to allow any modulation of venom composition (Figure 2c). One example is ants, which although they possess a long filamentous gland, store all venom in a contractile venom reservoir that is proximal to the venom delivery structures (as opposed to distal in cone snails). Although the Dufour’s gland could represent a secondary venom-producing structure, it appears that all toxins are transported to the venom reservoir, where they are stored—and expelled—together [115]. As a result, while able to quantitatively regulate venom secretion, ants appear to be unable to qualitatively modulate their venom due to morphological constraints, despite their venoms often playing a role in both predation and defense.

Other venomous lineages again are neither able to quantitatively nor qualitatively modulate their venom. Examples include most venomous fish lineages, which possess simple venom glands that produce relatively few venom components that are not differentially or serially secreted (Figure 2c) [115,116]. Even in the more complex of these venom apparatuses, such as the syringe-like structures of *Thalassophryne* spp. (venomous toadfishes) [117] or the voluminous glands of *Synanceia* spp. (stonefishes) [118], venom expulsion is driven directly by the process of wound infliction. In these cases, the venom producing tissue is either directly embedded in the victim (e.g., the barbs and spines of stingrays, chimaeras) or acts as a venom reservoir that can only be emptied passively, that is, by contact with the victim (e.g., toadfish, stonefish). This lack of ability to regulate the secretion of venom is probably also a reflection of the relative selection pressures associated with defensive *versus* predatory use of venom—while failure to capture prey likely only incurs a metabolic cost, failure to deter a predator results in immediate death. There is thus little room for evolving mechanisms of behavioral control over defensive venom secretions. In support of this observation, defensive uses of venom appear to be more of an all-or-nothing affair, even in animals with both predatory and defensive uses of their venom and that are able to quantitatively and/or qualitatively modulate their venom, such as rattlesnakes and cone snails, respectively [81,119]. However, while the majority of these venoms only play a role in defense (Table 2), many of the species that harbor them are preyed upon by a wide range of predators and the venoms are thus arguably multifunctional in terms of the need to induce pain in a wide range of organisms.

Animals with centralized venom systems can thus be roughly divided into three functional categories: (1) Species with very complex venom glands and a complex/diverse mixture of venom components, and which are very likely to be able to directly modulate venom secretion (Figure 2e). (2) Species with morphologically relatively simple glands but a high diversity of venom components that are stored heterogeneously throughout the gland, and which are likely able to achieve venom modulation indirectly by metering the amount of venom secreted (Figure 2d). (3) Species with a very simple venom gland morphology and only a few different venom components, and which are not likely to be able to modulate venom secretion at all (Figure 2c). It has to be noted, however, that the venom delivering structures of only very few animal lineages have been examined in detail, and that the actual mechanism of how venom is injected remains unknown for the majority of lineages. Clearly, more research needs to be conducted on morphological and behavioral aspects of venom biology to complete the picture of venom modulation across the animal kingdom. Nevertheless, venom gland morphology is likely to impose strong evolutionary constraints on the ability and type of venom modulation, and hence the functional and molecular evolution of its toxins.

## 5. Ecological Function and Venom Complexity

A commonly assumed relationship between function and toxin evolution is that predatory venoms evolve to become more complex than defensive venoms [23]. This relationship is based on comparisons of classic examples of defensive venoms such as those in fish and bees with well-known examples of venoms used for predation such as those in cone snails, spiders, and snakes—often collected by means of dissection, or electrical or manual stimulation. Defensive venoms often cause strong localized pain, which honey bee venom achieves almost exclusively by melittin (which accounts for ~80% of the venom) [132], and venoms from most fish achieve by a relatively simple and highly conserved cocktail [118]. In contrast, species that use their venoms for predation show a broad range of toxicity phenotypes (albeit usually measured in ecologically non-relevant models), and their venoms can be extremely complex, with hundreds to over a thousand unique venom components [133,134]. This difference in complexity is broadly considered to be due to defensive venoms evolving under negative selection to maintain their pain-inducing potency, while predatory toxins are engaged in a predator-prey arms race with their molecular targets in prey, which are constantly under selection to evolve resistance (see [14,135]). However, although a comprehensive review of the ecological factors that influence the composition of venoms is beyond the scope of this review, it appears that this relationship is perhaps not quite as straightforward due to the influences of multifunctionality, behavior, morphology of the venom system, and of course, how the studied venom has been collected.

Although predatory venoms often comprise diverse cocktails of toxins, their molecular diversities can differ substantially. For example, while the venoms of some spiders such as members of the funnel-web spider genus *Hadronyche* (Hexathelidae) may contain over a thousand unique venom components [133], this diversity does not appear to be universal across spiders but is dependent on the degree of specialization on particular prey [136]. Dietary breadth also appears to have an effect on the structural diversity contained within cone snail venoms [137,138], and is known to have a streamlining effect on venoms of snakes [139,140]. In addition, the venoms of centipedes may differ in complexity by an order of magnitude [10,141,142], including between species that are considered opportunistic generalist predators that feed on a wide range of prey. Lastly, the jointly predatory and defensive venoms from ants are simple mixtures that may consist of as little as less than 20 unique peptide and protein toxins [115].

In addition to the variability in venom complexity found across venomous lineages with predominantly predatory venoms, most of these species also use their venom for defense against predators (Figure 1). However, to what degree the increased molecular complexity of many predatory compared to defensive venoms is due to a difference versus an increase in their ecological function(s) remains largely unknown. It is also intriguing that the defensive venoms of cone snails appear to be more complex than their predatory venoms. In both *Conus geographus* and *C. marmoreus*, the components unique to the defensive venoms account for 44.7% and 66.7% of the total toxin diversity, respectively, compared to a contribution of 25% and 32.1% from the corresponding exclusively predatory toxins [95]. Thus, while predatory venoms may perhaps often be more complex than defensive venoms, it is not necessarily a universal phenomenon, and could even in part be a reflection of the increased number of ecological roles played by many predatory compared to purely defensive venoms.

It is also worth pointing out that there are additional, non-ecological factors that may drive or constrain the evolution of the complexity of venoms, such as various aspects of venom system morphology. For instance, the level of cellular complexity of venom glands has been proposed to constrain the molecular evolution of venom in centipedes [112]. Venom system morphology is also likely to determine if venom can be qualitatively modulated and hence to what degree venom components evolve as distinct functional groups that increase overall complexity (see Section 4). Another important aspect is the toxin mode of action, which may explain the lack of toxin diversity in both the defensive and predatory venoms of ants (see Section 6) [115]. Perhaps most important, however, is to improve our knowledge of the ecological and behavioral aspects of venom use. For example, we know very little about just how reliant different animals are on their venoms for predation and/or defense, or whether different feeding strategies have an effect on venom complexity.

## 6. Functional Diversity through Toxin Multi-Functionality

An assumption underlying the apparent conflict between the multifunctionality of venom and its optimization for a particular purpose is that different toxins perform different roles. Similarly, any relationship between venom complexity and ecological function(s) relies on there being a functional requirement of pharmacological diversity that is correlated with toxin diversity. However, toxins may achieve multiple functions in several ways, such as by specifically affecting ubiquitous targets, affecting targets with different roles in different animals, or by affecting many targets through pharmacological promiscuity. An obvious question is therefore whether there exists a relationship between the ability of an animal to qualitatively modulate its venom and the degree of multifunctionality of its toxins, and if so, whether the morphology of a venom system can be used to generate useful predictions about the pharmacological properties of the toxins contained in its venom (Figure 2a). Unfortunately, as mentioned previously, few venomous lineages have had their venom system morphology described in adequate detail to make predictions on venom modulation abilities, and even fewer have had both their toxins and venom apparatus described in detail.

### 6.1. Target Ubiquity

Examples of lineages with multifunctional venoms with relatively well-described toxin pharmacology and venom system morphology include cone snails, spiders, scorpions, ants, and snakes. Among these, however, only two lineages have been shown conclusively to qualitatively modulate (cone snails) or not modulate (ants) their venom. The differences between the venoms of these two lineages are striking. Although both are dominated by peptide toxins, cone snail venom almost exclusively consists of neurotoxic peptides (conotoxins) that comprise an incredible structural diversity spanning at least 22 superfamilies, and many of which can be grouped into distinct predatory and defensive functional categories [143]. In contrast, ant venoms are dominated by a single superfamily of membrane-interacting, mostly linear peptides called Aculeatoxins [115,144]. Aculeatoxins, as the name suggests, also appear to dominate the venoms of other aculeate hymenopterans, and include the hugely pharmacologically promiscuous toxins such as Melittin and Mastoparan, suggesting their largely membrane-targeting mode of action is well-suited to multifunctionality.

It is tempting to speculate that membrane-targeting toxins, as opposed to neurotoxic peptides, provide a pharmacological solution to provide multifunctionality in animals without the ability to qualitatively modulate their venom. After all, cell membranes on a whole probably represent the most ubiquitous of potential toxin targets. Reflecting this ubiquity, membrane-interacting toxins such as phospholipases and pore-forming toxins are exceedingly common throughout the toxinological universe [145]. They are not just found in venomous animals that lack the ability to qualitatively modulate their venom but also in venoms of assassin bugs, which are able to directly modulate the composition of their venom. Pore-forming toxins are also exceedingly diverse, and range from 2 kDa peptides, such as the smaller Aculeatoxins, to enormous multimeric proteins such as Stonustoxins [118], and we generally know little about their selectivity across biological membranes. For example, different Aculeatoxins show stark differences in their membrane affinities and interactions, resulting in a range of different cell type selectivities and pharmacological properties [115]. How this affects their ability to perform different functions, however, remains unknown.

### 6.2. Target Promiscuity

Another means of achieving multiple functions is by affecting multiple targets. Neurotoxic venom peptides are often held up as examples of bioactive molecules with incredible potency and exquisite selectivity, which have evolved and been refined into molecular scalpels for a single biological target over millions of years. This view may be partly correct for many toxins tested in specific model systems, where they may indeed provide amazingly selective pharmacological tools for dissecting physiological pathways. However, without diminishing the potential value of toxins as sources of new molecular tools, pesticides, and human therapeutics, it certainly does not hold for all (or perhaps even most) toxins, be they peptides or proteins.

This seems particularly evident for spider and scorpion venom gating-modifier toxins that target the voltage-sensing domains (VSDs) of voltage-gated ion channels (VGICs). Since they were first characterized, many of these toxins were observed to modulate multiple families of VGICs. For example, the spider toxins GrTX1 and Hanatoxin were originally characterized as voltage-gated calcium and potassium channel inhibitors, respectively. However, both were subsequently shown to inhibit both families of channels [146]. This observation has now been repeated many times with the most striking cases being the spider Protoxins from *Thrixopelma pruiriens* [147], and five peptides from *Grammostola spatulata* [148] that modulate a variety of voltage-gated sodium, potassium, and calcium channels. The ability of a given toxin to target multiple voltage sensors is also noted within channel subtypes and can result in two distinct pharmacological outcomes. For example, several tarantula toxins have been found to inhibit the VSDs in Nav domains I, II and III, resulting in inhibition of channel function, as well as targeting the domain IV VSD and inhibiting channel inactivation, thus enhancing channel function [149,150]. This dual pharmacology for a single toxin can contribute strongly to the multifunctionality of a venom. That is, both functions can play a synergistic role in causing prey paralysis (contributing to flaccid or spastic paralysis), while the inhibition of inactivation of Na_V_s in the nociceptors of predators would lead to intense pain, and thus play an import role in defense. Given the structural similarity of voltage-sensing domains of Na_V_s, Ca_V_s and K_V_s, and even TRPVs, it is not so surprising that venom peptides target multiple members and this seems to be the rule rather than the exception [149].

In contrast to the above examples where toxin promiscuity comes from targeting a relatively conserved structural motif, there are more and more examples emerging of toxins with truly diverse pharmacology spanning unrelated membrane proteins. The tarantula toxin GSMTx4 was originally isolated based on its ability to inhibit mechanosensitive channels, and subsequently shown to inhibit voltage-gated sodium and potassium channels in the 7–14 µM range [148] as well as possess antimicrobial activity in 0.5–30 µM range [151]. Likewise, the sea anemone peptide APETx2 was isolated as a selective inhibitor of the acid-sensing ion channel (ASIC) 3 [152], but has now been shown to also modulate ASIC 1b and 2a [153], several Na_V_ channels [154], as well as hERG (K_V_11.1) [155], thus modulating unrelated voltage- and ligand-gated ion channels in a similar concentration range. Recently, several cone snail ConoRFamides have also been found to have promiscuous activity, potentiating the activity of mammalian acid-sensing ion channels, but inhibiting muscle-type and neuronal nicotinic receptors [156]. In these examples, the pharmacological promiscuity has been determined while studying predominantly mammalian receptors, which may not represent ecologically relevant targets in terms of prey capture, but maybe have relevance in terms of a defensive function.

Unfortunately, this lack of availability or interest in ecologically relevant targets presents a major hurdle in attempting to generate an understanding of the pharmacological and taxonomic selectivity of toxins. Venoms may show substantial differences in potency between prey [157], and this may even be the case for individual toxins [20]. Indeed, we do not know the ecological relevance of many of the above examples of target promiscuity, and it is therefore not yet possible to conclude if the penchant of toxins for hitting multiple targets contributes more to synergism or to making a multipurpose venom in circumstances where venom gland morphology or innervation precludes bona fide venom modulation. Again, future studies that take a more holistic and ecologically-relevant approach will provide answers to these questions.

## 7. Conclusions

Venom systems have evolved on at least 100 independent occasions, spread across at least eight animal phyla. This enormous diversity of venomous animals means venoms are excellent models for studying questions in evolutionary biology through comparative methods, and at the same time represent a rich source of novel molecular tools and therapeutic and agrochemical leads. It is clear that despite decades of research, there is still much to learn about the overall function of venoms and their components. While the majority of venomous lineages use their venoms primarily to facilitate prey capture or feeding, venoms play a wide range of other roles and are used for multiple purposes by the same animal. How an animal achieves this multi-functionality depends to a large degree on the functional morphology and behavioral aspects of its venom system, which likely represent important factors that drive or constrain the functional evolution of their toxins. Thus, in order to gain a better understanding of venom biology, one needs to approach venoms as the integrated traits they really are and consider not just venom activity at the molecular target level, but function, the morphology of the venom apparatus, as well as behavioral aspects of venom delivery.

## Figures and Tables

**Table 1:** Functional diversity of venom. Examples of uses of venom beyond predation, defense, and blood-feeding.

Function	Example of Venomous Animal	References
Intraspecific competition	Platypus, sea anemones, slow loris	[51,52,53]
Food storage	Moles, shrews, parasitoid wasps	[54,55]
(Pre-)Digestion	Sea anemones, assassin bugs, centipedes, remipedes, vipers	[56,57,58,59,60]
Offspring care	Sea anemones, cubozoan jellyfish, parasitoid wasps, saw flies	[55,61,62,63]
Mating	Scorpions	[27]
Habitat creation	Ants	[64]
Antimicrobial ointment	Ants, wasps	[65]
Ectoparasite deterrent	Slow loris	[66]
Antivenom	Tawny crazy ant (*Nylanderia fulva*)	[28]
Prey homing device	Rattlesnakes	[67]
Intraspecific communication	Ants, wasps	[68,69,70]

**Table 2:** Predictions of potential ability to modulate venom in some venomous lineages. Predictions were made based on known venom system anatomy and potential multi-functionality of venom and do not include the known examples from scorpions, assassin bugs, and cone snails. Lineages that use their venoms for both predation and defense are italicized, while lineages with purely defensive venoms are marked with an asterisk.

Animal group	General Venom System Morphology	Type of Modulation
*Coleoid cephalopods*	Two pairs of potential venom glands, injected through muscular salivary papilla [120].	Quantitative regulation, direct qualitative modulation.
*Tonnoid, muricid, and colubrariid snails*	One or two lobes in venom glands that open through common duct into buccal mass [120].	Quantitative regulation, potentially direct qualitative modulation.
Nemertea	Proboscis with venom secreting cells, but no direct injection apparatus [121].	Potential qualitative modulation by spatially heterogeneous toxin storage along proboscis.
Glycerid polychaetes	Toxin-producing “lappets” secreting venom into large muscular and glandular venom reservoir, which is presumably also involved in venom expulsion [114].	Quantitative regulation.
Leeches	Secretory cells dispersed along the buccal cavity in jawed leeches (Arhynchobdellida); presence of two paired salivary glands in jawless leeches (Glossiphoniidae) [122,123].	Quantitative regulation and direct qualitative modulation in Glossiphoniidae; only quantitative regulation in Arhynchobdellida.
*Robber flies* (Asiliidae)	Two pairs of venom glands secreting venom to a separate venom pump [124].	Quantitative regulation, direct qualitative modulation.
*Larval neuropterans*	Paired venom gland opening directly into the venom delivering canal of the jaws [125].	Quantitative regulation.
*Aculeate hymenoptera*	Filamentous glands, venom stored in large venom reservoir. Additional Dufour’s gland [115].	Quantitative regulation, possibly direct qualitative modulation if Dufour’s gland involved.
*Lepidopteran caterpillars	Various variations on venom gland-associated spines [126].	None.
Fleas	Single pair of salivary/venom glands [127].	Quantitative regulation.
*Centipedes*	Composite venom glands consisting of numerous “secretory units” that empty into a chitinous duct (“calyx”). In most giant centipedes (Scolopendromorpha), the calyx is greatly extended, with secretory units organized perpendicular to length of the gland. Heterogeneous toxin production [112].	Quantitative regulation in all, direct qualitative modulation in giant centipedes.
Remipedes	Venom glands secrete into large venom reservoir immediately proximal to venom delivery structure [128].	Quantitative regulation.
*Spiders*	Paired muscular venom glands with branch-like ductules leading to a common duct. Spitting spiders (Scytodidae) with extra lobe.	Indirect qualitative modulation; direct qualitative modulation in spitting spiders.
Iocheiratan pseudoscorpions	Venom glands in pedipalpal fingers, either in both, or in either, with separate outlets [129].	Quantitative regulation, potential direct qualitative modulation in species with venom glands in both pedipalpal fingers.
*Echinoderms	Venomous spines, venomous pedicellaria [130,131].	None. Potential spatial heterogeneity of toxins with different functions.
*Fish, except lampreys, fang blennies, and jaw eels	Venomous spines connected to or covered in venom-producing glands/tissue.	None.
*Frogs, salamanders	Spines or ribs piercing venom glands.	None.
*Colubroid snakes*	Venom glands with branch-like ductules leading to a short duct connected to front or rear fangs.	Quantitative regulation, indirect qualitative modulation.

## Supplementary Materials

The following are available online at https://www.mdpi.com/2072-6651/11/11/666/s1, Table S1: Independently evolved venomous animal lineages and the primary ecological roles of their venoms. Taxa for which no direct support of their venomous nature could be found are shown in grey font.
