# Supplementary material for: The Diversity of Venom: The Importance of Behavior and Venom System Morphology in Understanding Its Ecology and Evolution
Source: Toxins (Basel). 2019 Nov 14;11(11):666. doi: 10.3390/toxins11110666 (PMC6891279; doi:10.3390/toxins11110666)
Supplement: Supplementary file 1 [file toxins-11-00666-s001.pdf]

Supplementary Materials: The Diversity of Venom: The Importance of Behavior and Venom System Morphology in Understanding Its Ecology and Evolution

Vanessa Schendel, Lachlan D. Rash, Ronald A. Jenner, and Eivind A. B. Undheim

Table S1. Independently evolved venomous animal lineages and the primary ecological roles of their venoms. Taxa for which no direct support of their venomous nature could be found are shown in grey font.

Animal Group	Venomous Lineage	General Venom System Morphology	Primary Role	References
Cnidarians	All	Nematocysts	Predation, defense, intraspecific competition	[1]
Molluscs	Coleoid cephalopods, including octopus and squid	Posterior and anterior glands, venom injected through salivary papilla.	Predation	[2,3]
	Cone snails and relatives (Conoidea)	Long duct/venom gland, venom injected through hollow radular tooth on proboscis by a distal venom pump.	Predation, defense	[4]
	Tritons, helmet shells, etc. (Tonnoidea)	Two-lobed salivary (venom) glands that open through common duct into buccal mass.	Predation	[5]
	Dwarf tritons, including vampire snails (Colubrariidae)	Single-lobed salivary (venom) glands that open through common duct into buccal mass.	Predation	[6]
	Murex snails (Muricidae)	Primary and accessory salivary (venom) glands that open through common duct into buccal mass.	Predation	[7]
Nemerteans	Ribbon worms	Proboscis with venom secreting cells, sometimes with stylet to facilitate venom delivery (Enopla), or pseudocnidae with a potential role in venom delivery.	Predation	[8]
Annelids	Blood worms (Glyceridae)	Toxin-producing “lappets” secreting venom into large muscular and glandular venom reservoir, which is presumably also involved in venom expulsion.	Predation	[9]
	Leeches (Hirudinea)	Secretory cells dispersed along the buccal cavity in jawed leeches (Arhynchobdellida);	Predation, blood feeding	[10–12]

		presence of two paired salivary glands in jawless leeches (Glossiphoniidae).		
Flatworms	Genus *Prorhynchus*	Gland cells connected to a hollow stylet that functions as penis in mature individuals.	Predation	[13]
	Genus *Mesostoma*	Unknown.	Predation	[14]
Arthropods Insects	Sandflies (Phlebotominae)	Salivary glands connected to proboscis.	Blood feeding	[15,16]
	Midges (Ceratopogonidae)	Salivary glands connected to proboscis.	Blood feeding	[16,17]
	Black flies (Simuliidae)	Salivary glands connected to proboscis.	Blood feeding	[16,18,19]
	Mosquitoes (Culicidae)	Salivary glands connected to proboscis.	Blood feeding	[16,20,21]
	Biting midges (Corethrellidae)	Salivary glands connected to proboscis.	Blood feeding	[16,22]
	Snipe flies (Rhagionidae)	Salivary glands connected to proboscis.	Blood feeding, predation	[16]
	Ibis flies (Athericidae)	Salivary glands connected to proboscis.	Blood feeding	[16]
	Horseflies (Tabanidae)	Salivary glands connected to proboscis.	Blood feeding	[16,23]
	Horsefly larvae (Tabanidae)	Venom injected through hollow mandibles.	Predation	[24]
	Frit flies (e.g., *Batrachomyia* spp.)	Salivary glands connected to proboscis.	Blood feeding	[16]
	Bat flies (Streblidae)	Salivary glands connected to proboscis.	Blood feeding	[16]
	Tsetse flies, louseflies and allies (Calyptrata)	Salivary glands connected to proboscis.	Blood feeding	[16,25,26]
	Garbage fly larvae (*Hydrotaea leucostoma*, Muscidae)	Salivary glands connected to proboscis.	Predation	[27]
	Stablefly (*Stomoxys* spp., Muscidae)	Salivary glands connected to proboscis.	Blood feeding	[28]
	Cheesefly larvae (Piophilidae)	Venom injected through hollow mandibles, venom glands remain unknown.	Blood feeding	[16]
	Grassfly larvae (Chloropidae)	Unknown	Blood feeding	[16]
	Bird flies (Carnidae)	Salivary glands connected to proboscis.	Blood feeding	[16]
	Hoverfly larvae (Syrphidae)	Unknown	Predation	[24]

Robber flies (Asiliidae)	Two pairs of venom glands secreting venom to a venom pump that injects venom through the proboscis.	Predation	[16]
Slug-eating fly larvae (Sciomyzidae)	Venom produced in salivary glands and injected through hollow mouth hooks.	Predation	[29]
Gall gnat larvae (Cecidomyiidae)	Venom produced in salivary glands and injected through hollow mandibles.	Predation	[24]
Diving beetles and allies (Adephaga)	Venom injected through hollow mandibles, details of venom production remains unknown.	Predation	[24,29]
Rove beetles and allies (Staphylinoidea, Hydrophiloidea)	Venom injected through hollow mandibles, details of venom production remains unknown.	Predation	[24,29]
Fireflies, glow-worm beetles and allies (Lampyridae, Phengodidae)	Venom injected through hollow mandibles, details of venom production remains unknown.	Predation	[24,29,30]
Scorpion beetle (*Onychocerus albitarsis*)	Terminal antennal segment modified into a venom gland-containing stinger that closely resembles that of a scorpion.	Defense	[31]
Sucking lice (Anoplura)	Salivary glands connected to proboscis.	Blood feeding	[32]
Fleas (Siphonaptera)	Salivary glands connected to proboscis.	Blood feeding	[33,34]
Eusocial aphid soldiers (Pemphigidae)	Venomous soldier caste. Venom produced in uncharacterised glands, injected with stylet.	Defense	[35,36]
Eusocial aphid soldiers (Hormaphididae)	Venomous soldier caste. Venom produced in uncharacterised glands, injected with stylet.	Defense	[36,37]
Heteroptera	Up to four-lobed main venom gland and accessory gland connected via a hilus to venom pump and injected through a venom canal in the proboscis.	Predation, defense	[38]
Predatory stinkbugs (Asopinae)	Venom and accessory gland connected via a hilus to venom pump and injected through a venom canal in the proboscis.	Predation	[38]
Big-eyed bugs (Geocoridae)	Venom and accessory gland connected via a hilus to venom pump and injected through a venom canal in the proboscis.	Predation	[38]

	Capsid bugs (Miridae)	Venom and accessory gland connected via a hilus to venom pump and injected through a venom canal in the proboscis.	Predation	[38]
	Predatory red bugs (*Antilochus* spp. and *Raxa* spp.)	Venom and accessory gland connected via a hilus to venom pump and injected through a venom canal in the proboscis.	Predation	[38]
	Lacewing larvae and allies (Neuroptera)	Paired venom glands each connected to "fangs" made from fused maxillae and mandibles.	Predation	[24]
	Wasps and relatives (Hymenoptera)	Thin filamentous gland secreting toxins into venom reservoir connected through a venom duct to posterior stinger that is a modified ovipositor.	Predation, defense	[24,29]
	Cup moths (Limacodidae)	Venomous spines lined with hypodermal toxin-producing cells.	Defense	[39]
	Pine-tree lappet moth (*Dendrolimus pini*)	In addition to urticating hairs, have hollow spines with toxin-producing epithelial cell clusters at the base.	Defense	[39]
	Processionary tree caterpillars (Notodontidae)	In addition to urticating hairs that can be released by minimal mechanical stimuli and carried by wind, have non-removable spines with unknown toxin-producing cells.	Defense	[39]
	Flannel moths (Megalopygidae)	Hollow spines, with venom presumed produced by basal cells and contained within the spines.	Defense	[39]
	Tiger moths (Arctiinae)	Tufts of urticating hairs. Modified, balloon-like setae with venom glands at the base (*Lymantria dispar*).	Defense	[39]
	Buck moths, including *Lonomia* spp. (Hemileucinae)	Tufts of venom-bearing spines (scoli).	Defense	[39]
	Termite soldiers (*Armitermes* and *Cubitermes*)	Venom applied through "snout" to wound inflicted by mandibles.	Defense	[40]
Arthropods Crustaceans	Salmon lice (Copepoda, Siphonostomatoida)	Unknown	Blood feeding	[41,42]
	Cyclopoid copepods (*Lernaea* spp.)	Unknown	Blood feeding	[42]

	Venomous copepods (*Heterorhabdus* and relatives)	Unknown venom producing tissue connected to presumed hollow maxillae.	Predation	[43]
	Carp lice (Branchiura)	Venom glands connected to venom delivering stylet.	Blood feeding	[42,44]
	Remipedes (Remipedia)	Venom glands secrete into large venom reservoir immediately proximal to venom delivery structure	Predation	[45,46]
	Skeleton shrimp (Amphipoda, Caprellidae)	Unknown venom glands producing toxins delivered through "thumb" on gnathopod. Male specific.	Intraspecific competition	[47]
	Predatory hyperiid amphipod (*Primno* spp, possibly others in Phronimoidea)	Large, two-lobed gland in pereopod 2 (possibly pereopods 5–7 in other genera) that connects to a pore at the tip of the dactyl.	Predation	[48]
	Gnathiid isopods (Gnathiidae)	Unknown	Blood feeding	[42,49]
	Fish lice (Isopoda, Cymothoidae)	Unknown	Blood feeding	[42]
Arthropods Chilopods	Centipedes (Chilopoda)	Composite venom glands consisting of numerous "secretory units" that empty into a chitinous duct ("calyx"). In most giant centipedes (Scolopendromorpha), the calyx is greatly extended, with secretory units organized perpendicular to length of the gland. Heterogeneous toxin production.	Predation, defense	[50,51]
Arthropods Arachnids	Pseudoscorpions	Venom glands in pedipalpal fingers, either in both, or in either, with separate outlets.	Predation	[52,53]
	Spiders	Paired muscular venom glands with branch-like ductules leading to a common duct. Spitting spiders (Scytodidae) with extra lobe.	Predation, defense	[54]
	Predatory mites and ticks	Venom produced in salivary glands.	Predation, blood feeding	[55-58]
	Scorpions	Venom gland with paired lobes in telson, each with branched ductules leading to a small	Predation, defense	[59]

		lumen connected to stinger through short duct.		
Echinoderms	Crown of thorns (Acanthasteridae)	Spines covered in venom-producing tissue.	Defense	[60,61]
	Leather/Fire urchins, hollow-spined sea urchins (Echinothurioida and Diadematoida), venomous spines	Spines covered in venom-producing tissue.	Defense	[62,63]
	Sea urchins (Echinoida), venomous pedicellariae	Venom-producing pedicellariae.	Defense	[62,63]
Chaetognaths	Arrow worms (Chaetognatha)	Unknown	Predation	[64]
Chordates	Lampreys	Venom produced in salivary glands and delivered during feeding.	Blood feeding	[65,66]
	Chimaeras (Chimaeriformes)	Dorsal spine with grooves lined with venom-producing tissue.	Defense	[67]
	Stingrays and relatives (Myliobatiformes)	Barbed caudal spine partly covered in venom-producing tissue.	Defense	[67]
	Hornsharks (Heterodontidae)	Dorsal spine partly covered in venom-producing tissue	Defense	[67]
	Dogfish (Squalidae)	Dorsal spine partly covered in venom-producing tissue.	Defense	[67]
	Catfish (Siluroidea)	Dorsal and pectoral spines partly covered in venom-producing tissue.	Defense	[67]
	Toadfishes (Thalassophryninae)	Dorsal and opercular spines with venom gland connected to the base of the hollow spines through which venom is injected.	Defense	[67]
	Stargazers (Uranoscopidae)	Opercular spines partly covered in venom-producing tissue.	Defense	[67]
	Weeverfishes (Trachinidae)	Dorsal spine partly covered in venom-producing tissue.	Defense	[67]
	Fang blennies (genus *Meiacanthus*, family Blenniidae)	Paired fangs in lower jaw connected to venom glands.	Defence? Predation? Intraspecific competition?	[67]
	Jaw eels (Monognathidae)	Rostral fang connected to undescribed venom-producing tissue.	Predation	[67]
	Jacks (Scomberoidinae)	Dorsal spine partly covered in venom-producing tissue.	Defense	[67]

Gurnard perch (genus *Neosebastes*)	Dorsal spine partly covered in venom-producing tissue.	Defense	[67]
Scorpion fish (Scorpaenidae, Sebastidae, Setarchidae)	Dorsal spine partly covered in venom-producing tissue.	Defense	[67]
Stonefishes and wasp fishes (some Aploactinidae, Apistidae, Eschmeyeridae, Gnathanacanthidae, Synanceiidae, Tetrarogidae)	Dorsal, pectoral, and anal fin spines with large venom glands opening to tip of spine through groove.	Defense	[67]
Surgeonfishes (some acanthurids)	Dorsal, anal, and pelvic spines with venom glands.	Defense	[67]
Rabbitfishes (Siganidae)	Dorsal, anal, and pelvic spines with venom glands.	Defense	[67]
Scats (Scatophagidae)	Dorsal spine partly covered in venom-producing tissue.	Defense	[67]
Clingfishes (some gobiesocines)	Subopercular spine with venom gland.	Defense	[67]
Hylid frogs (*Corythomantis greening, Aparasphenodon brunoi*)	Venom delivered by cranial spines that pierce through venom producing glands on skin.	Defense	[68]
Spiny newts (genus *Echinotriton*)	Venom delivered by sharp ribs piercing through venom producing glands on skin.	Defense	[69]
Ribbed newts (genus *Pleurodeles*)	Venom delivered by sharp ribs piercing through venom producing skin glands.	Defense	[70]
Shrews (Soricidae, *Neomys*)	Venom produced in enlarged and granular submaxillary salivary glands.	Predation	[71]
Shrews (Soricidae, *Blarina*)	Venom produced in enlarged and granular submaxillary salivary glands.	Predation	[71]
Moles (Talpidae)	Venom produced in enlarged and granular submaxillary salivary glands.	Predation	[71]
Solenodons (Solenodontidae)	Venom produced in enlarged and granular submaxillary salivary glands at the base of modified lower second incisors with deep groove.	Predation	[71]

Slow lorises (genus *Nycticebus*)	Venom probably produced in brachial and submaxillary glands.	Defense or intraspecific competition	[71,72]
Vampire bats (Desmodontinae)	Venom produced in principal submaxillary gland.	Blood feeding	[70,72]
Platypus (*Ornithorhynchus anatinus*)	Crural gland connected to spur on hind leg through long canal. Only in males and during mating season.	Intraspecific competition	[73]
Colubroid snakes	Venom glands with branch-like ductules leading to a short duct connected to front or rear fangs in upper jaw. In many elapids and vipers, duct runs through an accessory gland of unknown function.	Predation, defense	[74,75]
Varanid lizards	Venom gland in lower jaw, delivered through grooved teeth.	Predation, defense	[74,75]
Helodermatid lizards	Venom gland in lower jaw, delivered through grooved teeth.	Defense	[74,75]
Total number of independently evolved venomous lineages (conservative estimate)			104 (101)

References

1. Jouiaei, M.; Yanagihara, A.A.; Madio, B.; Nevalainen, T.J.; Alewood, P.F.; Fry, B.G. Ancient venom systems: A review on Cnidaria toxins. *Toxins* **2015**, *7*, 2251–2271.
2. Fry, B.G.; Roelants, K.; Norman, J.A. Tentacles of venom: toxic protein convergence in the kingdom Animalia. *J. Mol. Evol.* **2009**, *68*, 311–321.
3. Fingerhut, L.; Strugnell, J.M.; Faou, P.; Labiaga, A.R.; Zhang, J.; Cooke, I.R. Shotgun proteomics analysis of saliva and salivary gland tissue from the common octopus *Octopus vulgaris*. *J. Proteome Res.* **2018**, *17*, 3866–3876.
4. Prashanth, J.R.; Dutertre, S.; Lewis, R.J. Pharmacology of predatory and defensive venom peptides in cone snails. *Mol. Biosyst.* **2017**, *13*, 2453–2465.
5. Bose, U.; Wang, T.; Zhao, M.; Motti, C.A.; Hall, M.R.; Cummins, S.F. Multiomics analysis of the giant triton snail salivary gland, a crown-of-thorns starfish predator. *Sci. Rep.* **2017**, *6*, 6000.
6. Modica, M.V.; Lombardo, F.; Franchini, P.; Oliverio, M. The venomous cocktail of the vampire snail *Colubraria reticulata* (Mollusca, Gastropoda). *BMC Genomics* **2015**, *16*, 441.
7. Modica, M.V.; Holford, M. The evolutionary innovations of predatory marine snails with remarkable pharmacological potential. In *Evolutionary biology—Concepts, molecular and morphological evolution*, Pontarotti, P., Ed. Springer-Verlag: Berlin, 2010; pp 249–270.
8. Göransson, U.; Jacobsson, E.; Strand, M.; Andersson, H.S. The toxins of nemertean worms. *Toxins* **2019**, *11*, 120.
9. Richter, S.; Helm, C.; Meunier, F.A.; Hering, L.; Campbell, L.I.; Drukewitz, S.H.; Undheim, E.A.B.; Jenner, R.A.; Schiavo, G.; Bleidorn, C. Comparative analyses of glycerotoxin expression unveil a novel structural organization of the bloodworm venom system. *BMC Evol. Biol.* **2017**, *17*, 1–19.
10. Marshall, C.G.; Lent, C.M. Excitability and secretory activity in the salivary gland cells of jawed leeches (Hirudinea: Gnathobdellida). *J. Exp. Biol.* **1988**, *137*, 89–105.
11. Moser, W.E.; Desser, S.S. Morphological, histochemical, and ultrastructural characterization of the salivary glands and proboscises of three species of glossiphoniid leeches (Hirudinea: Rhynchobdellida). *J. Morphol.* **1995**, *225*, 1–18.
12. Tessler, M.; Marancik, D.; Champagne, D.; Dove, A.; Camus, A.; Siddall, M.E.; Kvist, S. Marine leech anticoagulant diversity and evolution. *J. Parasitol.* **2018**, *104*, 210–220.

13. Tyler, S.; Varjabedian, A.; Hamami, E. Functional morphology of the venom apparatus of *Prorhynchus stagnalis* (Platyhelminthes, Lecithoepitheliata). *Zoomorphology* **2017**, *137*, 19–29.
14. von Reumont, B.M.; Campbell, L.I.; Jenner, R.A. *Quo Vadis v*enomics? A roadmap to neglected venomous invertebrates. *Toxins* **2014**, *6*, 3488–3551.
15. Lestinova, T.; Rohousova, I.; Sima, M.; de Oliveira, C.I.; Volf, P. Insights into the sand fly saliva: blood-feeding and immune interactions between sand flies, hosts, and *Leishmania*. *PLoS Negl. Trop. Dis.* **2017**, *11*, e0005600.
16. Wiegmann, B.M.; Trautwein, M.D.; Winkler, I.S.; Barr, N.B.; Kim, J.-W.; Lambkin, C.; Bertone, M.A.; Cassel, B.K.; Bayless, K.M.; Heimberg, A.M., *et al.* Episodic radiations in the fly tree of life. *Proc. Natl. Acad. Sci. USA* **2011**, *108*, 5690–5695.
17. Lehiy, C.J.; Drolet, B.S. The salivary secretome of the biting midge, *Culicoides sonorensis*. *PeerJ* **2014**, *2*, e426.
18. Chagas, A.C.; Calvo, E.; Pimenta, P.F.P.; Ribeiro, J.M.C. An insight into the sialome of *Simulium guianense* (Diptera: Simuliidae), the main vector of River Blindness Disease in Brazil. *BMC Genomics* **2011**, *12*, 612.
19. Hempolchom, C.; Reamtong, O.; Sookrung, N.; Srisuka, W.; Sakolvaree, Y.; Chaicumpa, W.; Taai, K.; Dedkhad, W.; Jariyapan, N.; Takaoka, H., *et al.* Proteomes of the female salivary glands of *Simulium nigrogilvum* and *Simulium nodosum*, the main human-biting black flies in Thailand. *Acta Trop.* **2019**, *194*, 82–88.
20. Arca, B.; Lombardo, F.; Struchiner, C.J.; Ribeiro, J.M. Anopheline salivary protein genes and gene families: an evolutionary overview after the whole genome sequence of sixteen Anopheles species. *BMC Genomics* **2017**, *18*, 153.
21. Chagas, A.C.; Calvo, E.; Rios-Velásquez, C.M.; Pessoa, F.A.; Medeiros, J.F.; Ribeiro, J.M. A deep insight into the sialotranscriptome of the mosquito, *Psorophora albipes*. *BMC Genomics* **2013**, *14*, 875.
22. Ribeiro, J.M.C.; Chagas, A.C.; Pham, V.M.; Lounibos, L.P.; Cavo, E. An insight into the sialome of the frog biting fly, *Corethrella appendiculata*. *Insect Biochem. Mol. Biol.* **2014**, *44*, 23–32.
23. Ribeiro, J.M.C.; Kazimirova, M.; Takac, P.; Andersen, J.F.; Francischetti, I.M.B. An insight into the sialome of the horse fly, *Tabanus bromius*. *Insect Biochem. Mol. Biol.* **2015**, *65*, 83–90.
24. Schmidt, J.O. Biochemistry of insect venoms. *Annu. Rev. Entomol.* **1982**, *27*, 339–368.
25. Alves-Silva, J.; Ribeiro, J.M.C.; Abbeele, J.; Attardo, G.; Hao, Z.; Haines, L.R.; Soares, M.B.; Berriman, M.; Aksoy, S.; Lehane, M.J. An insight into the sialome of *Glossina morsitans morsitans*. *BMC Genomics* **2010**, *11*, 213.
26. Bai, X.; Yao, H.; Du, C.; Chen, Y.; Lai, R.; Rong, M. An immunoregulatory peptide from tsetse fly salivary glands of *Glossina morsitans morsitans*. *Biochimie* **2015**, *118*, 123–128.
27. Anderson, J.R.; Poorbaugh, J.H. Biological control possibility for house flies. *Calif. Agric.* **1964**, *18*, 2–4.
28. Wang, T.; Zhao, M.; Rotgans, B.A.; Ni, G.; Dean, J.F.; Nahrung, H.F.; Cummins, S.F. Proteomic analysis of the venom and venom sac of the woodwasp, *Sirex noctilio* - Towards understanding its biological impact. *J. Proteomics* **2016**, *146*, 195–206.
29. Walker, A.A.; Robinson, S.D.; Yeates, D.K.; Jin, J.; Baumann, K.; Dobson, J.; Fry, B.G.; King, G.F. Entomo-venomics: the evolution, biology and biochemistry of insect venoms. *Toxicon* **2018**, *154*, 15–27.
30. Eisner, T.; Eisner, M.; Attygalle, A.B.; Deyrup, M.; Meinwald, J. Rendering the inedible edible: Circumvention of a millipede's chemical defense by a predaceous beetle larva (Phengodidae). *Proc. Natl. Acad. Sci. USA* **1998**, *95*, 1108–1113.
31. Berkov, A.; Rodriguez, N.; Centeno, P. Convergent evolution in the antennae of a cerambycid beetle, Onychocerus albitarsis, and the sting of a scorpion. *Naturwissenschaften* **2008**, *95*, 257–261.
32. Waniek, P.J. The digestive system of human lice: current advances and potential applications. *Physiol. Entomol.* **2009**, *34*, 203–210.
33. Andersen, J.F.; Hinnebusch, B.J.; Lucas, D.A.; Conrads, T.P.; Veenstra, T.D.; Pham, V.M.; Ribeiro, J.M.C. An insight into the sialome of the oriental rat flea, *Xenopsylla cheopis* (Rots). *BMC Genomics* **2007**, *8*, 102.
34. Ribeiro, J.M.C.; Assumpção, T.C.F.; Ma, D.; Alavarenga, P.H.; Pham, V.M.; Andersen, J.F.; Francischetti, I.M.B.; Macaluso, K.R. An insight into the sialotranscriptome of the cat flea, *Ctenocephalides felis*. *PLOS One* **2012**, *7*, e44612.
35. Lawson, S.P.; Sigle, L.T.; Lind, A.L.; Legan, A.W.; Mezzanotte, J.N.; Honegger, H.-W.; Abbot, P. An alternative pathway to eusociality: exploring the molecular and functional basis of fortress defense. *Evolution* **2017**, *71*, 1986–1998.
36. Stern, D.L.; Foster, W.A. The evolution of soldiers in aphids. *Biol. Rev. Camb. Philos. Soc.* **1996**, *71*, 27–79.

37. Kutsukake, M.; Nikoh, N.; Shibao, H.; Rispe, C.; Simon, J.C.; Fukatsu, T. Evolution of soldier-specific venomous protease in social aphids. *Mol. Biol. Evol.* **2008**, *25*, 2627–2641.
38. Walker, A.A.; Weirauch, C.; Fry, B.G.; King, G.F. Venoms of heteropteran insects: A treasure trove of diverse pharmacological toolkits. *Toxins* **2016**, *8*, 43.
39. Villas-Boas, I.M.; Bonfá, G.; Tambourgi, D.V. Venomous caterpillars: from inoculation apparatus to venom composition and envenomation. *Toxicon* **2018**, *153*, 39–52.
40. Chuah, C.H.; Goh, S.H.; Prestwich, G.D.; Tho, Y.P. Soldier defense secretions of the Malaysian termite, *Hospitalioermes umbrinus* (Isoptera, Nasutitermitinae). *J. Chem. Ecol.* **1983**, *9*, 347–356.
41. Øvergård, A.-C.; Hamre, L.A.; Harasimczuk, E.; Sussie, D.; Nilsen, F.; Grotmol, S. Exocrine glands of *Lepeophtheirus salmonis* (Copepoda: Caligidae): distribution, developmental appearance, and the site of secretion. *J. Morphol.* **2016**, *277*, 1616–1630.
42. Hadfield, K.A.; Smit, N.J. Parasitic Crustacea as vectors. In *Parasitic Crustacea*, Smit, N.J.; Bruce, N.; Hadfield, K., Eds. Springer, Cham: 2019.
43. Hirabayashi, T.; Ohtsuka, S.; Urata, M.; Tomikawa, K.; Tanaka, H. Molecular evidence on evolutionary switching from particle-feeding to sophisticated carnivory in the calanoid copepod family Heterorhabdidae: drastic and rapid changes in functions of homologues. *J. Nat. Hist.* **2016**, *50*, 1759–1772.
44. AmbuAli, A.; Monaghan, S.J.; Al-Adawi, K.; Al-Kindi, M.; Bron, J.E. Histological and histochemical characterisation of glands associated with the feeding appendages of *Argulus foliaceus* (Linnaeus, 1758). *Parasit. Int.* **2019**, *69*, 82–92.
45. von Reumont, B.M.; Blanke, A.; Richter, S.; Alvarez, F.; Bleidorn, C.; Jenner, R.A. The first venomous crustacean revealed by transcriptomics and functional morphology: remipede venom glands express a unique toxin cocktail dominated by enzymes and a neurotoxin. *Mol. Biol. Evol.* **2014**, *31*, 48–58.
46. Von Reumont, B.M.; Undheim, E.A.B.; Jauss, R.T.; Jenner, R.A. Venomics of remipede crustaceans reveals novel peptide diversity and illuminates the venom's biological role. *Toxins* **2017**, *9*, 234.
47. Takeshita, F.; Wada, S. Morphological comparison of the second gnathopod in males of four caprellid species (Amphipoda: Caprellidae). *J. Crust. Biol.* **2012**, *32*, 673–676.
48. Bowman, T.E. Revision of the pelagic amphipod genus *Primo* (Hyperiidea: Phrosinidae). *Smithson. Contrib. Zool.* **1978**, *275*, 1–23.
49. Manship, B.M.; Walker, A.J.; Jones, L.A.; Davies, A.J. Blood feeding in juvenile *Paragnathia formica* (Isopoda: Gnathiidae): biochemical characterization of trypsin inhibitors, detection of anticoagulants, and molecular identification of fish hosts. *Parasitology* **2012**, *139*, 744–754.
50. Undheim, E.A.; Fry, B.G.; King, G.F. Centipede venom: recent discoveries and current state of knowledge. *Toxins* **2015**, *7*, 679–704.
51. Undheim, E.A.B.; King, G.F. On the venom system of centipedes (Chilopoda), a neglected group of venomous animals. *Toxicon* **2011**, *57*, 512–524.
52. Krämer, J.; Pohl, H.; Predel, R. Venom collection and analysis in the pseudoscorpion *Chelifer cancroides* (Pseudoscorpiones: Cheliferidae). *Toxicon* **2019**, *162*, 15–23.
53. Santibáñez-López, C.E.; Ontano, A.Z.; Harvey, M.S.; Sharma, P.P. Transcriptomic analysis of pseudoscorpion venom reveals a unique cocktail dominated by enzymes and protease inhibitors. *Toxins* **2018**, *10*, 207.
54. Herzig, V.; King, G.F.; Undheim, E.A.B. Can we resolve the taxonomic bias in spider venom research? *Toxicon: X* **2019**, *1*, 100005.
55. Cabezas-Cruz, A.; Valdés, J.J. Are ticks venomous animals? *Frontiers in zoology* **2014**, *11*, 47.
56. Hoy, M.A.; Waterhouse, R.M.; Wu, K.; Estep, A.S.; Ioannidis, P.; Palmer, W.J.; Pomerantz, A.F.; Simão, F.A.; Thomas, J.; Jiggins, F.M., *et al.* Genome sequencing of the phytoseiid predatory mite *Metaseiulus occidentalis* reveals completely atomized Hox genes and superdynamic intron evolution. *Genome Biol. Evol.* **2016**, *8*, 1762–1775.
57. Lozano-Fernandez, J.; Tanner, A.R.; Giacomelli, M.; Carton, R.; Vinther, J.; Edgecombe, G.D.; Pisani, D. Increasing species sampling in chelicerate genomic-scale datasets provides support for monophyly of Acari and Arachnida. *Nat. Commun.* **2019**, *10*, 2295.
58. Zhang, Y.; Han, R. Insight into the salivary secretome of *Varroa destructor* and salivary toxicity to *Apis cerana*. *J. Econ. Entomol.* **2019**, *112*, 505–514.

59. Evans, E.R.J.; Northfield, T.D.; Daly, N.L.; Wilson, D.T. Venom costs and optimization in scorpions. *Front. Ecol. Evol.* **2019**, *7*, 1–7.
60. Hall, M.R.; Kocot, K.M.; Baughman, K.W.; Fernandez-Valverde, S.L.; Gauthier, M.E.A.; Hatleberg, W.L.; Krishnan, A.; Mcdougall, C.; Motti, C.A.; Shoguchi, E., *et al.* The crown-of-thorns starfish genome as a guide for biocontrol of this coral reef pest. *Nature* **2017**, *544*, 231–234.
61. Shiomi, K.; Midorikawa, M.; Nagashima, Y.; Nagai, H. Plancitoxins, lethal factors from the crown-of-thorns starfish *Acanthaster planci*, are deoxyribonucleases II. *Toxicon* **2004**, *44*, 499–506.
62. Coppard, S.E.; Kroh, A.; Smith, A.B. The evolution of pedicellariae in echinoids: an arms race against pests and parasites. *Acta Zool.* **2010**, *93*, 125–148.
63. Koch, N.M.; Coppard, S.E.; Lessios, H.A.; Briggs, D.E.G.; Mooi, R.; Rouse, G.W. A phylogenomic resolution of the sea urchin tree of life. *BMC Evol. Biol.* **2018**, *18*, 189.
64. Bieri, R.; Thuesen, E.V. The strange worm *Bathybelos*. *Am. Sci.* **1990**, *78*, 542–549.
65. Harris, R.J.; Jenner, R.A. Evolutionary ecology of fish venom: Adaptations and consequences of evolving a venom system. *Toxins* **2019**, *11*, 60.
66. Li, B.; Gou, M.; Han, J.; Yuan, X.; Li, Y.; Li, T.; Jiang, Q.; Xiao, R.; Li, Q. Proteomic analysis of buccal gland secretion from fasting and feeding lampreys (*Lampetra morii*). *Proteome Sci.* **2018**, *16*, 9.
67. Smith, W.L.; Stern, J.H.; Girard, M.G.; Davis, M.P. Evolution of venomous cartilaginous and ray-finned fishes. *Integr. Comp. Biol.* **2016**, *56*, 950–961.
68. Jared, C.; Mailho-Fontana, Pedro L.; Antoniazzi, Marta M.; Mendes, Vanessa A.; Barbaro, Katia C.; Rodrigues, Miguel T.; Jr, Edmund D.B. Venomous frogs use heads as weapons. *Curr. Biol.* **2015**, *25*, 2166–2170.
69. Brodie, J., Edmund D. ; Nussbaum, R.A.; DiGiovanni, M. Antipredator adaptations of Asian salamanders (Salamandridae). *Herpetologica* **1984**, *40*, 56–68.
70. Nowak, R.; Brodie Jr, E. Rib penetration and associated antipredator adaptations in the salamander *Pleurodeles waltl* (Salamandridae). *Copeia* **1978**, *3*, 424–429.
71. Rode-Margono, J.E.; Nekaris, K.A. Cabinet of curiosities: venom systems and their ecological function in mammals, with a focus on primates. *Toxins* **2015**, *7*, 2639–2658.
72. Nekaris, K.; Moore, R.; Rode, E.; Fry, B. Mad, bad and dangerous to know: the biochemistry, ecology and evolution of slow loris venom. *J. Venom. Anim. Toxins Incl. Trop. Dis.* **2013**, *19*, 21.
73. Whittington, C.M.; Belov, K. Tracing monotreme venom evolution in the genomics era. *Toxins* **2014**, *6*, 1260–1273.
74. Fry, B.G. *Venomous reptiles & their toxins. Evolution, pathophysiology & biodiversity*. Oxford University Press: Oxford, 2015.
75. Hargreaves, A.D.; Swain, M.T.; Logan, D.W.; Mulley, J.F. Testing the Toxicofera: Comparative transcriptomics casts doubt on the single, early evolution of the reptile venom system. *Toxicon* **2014**, *92*, 140–156.
